# Epicardial fat volume is inversely correlated with the degree of diastolic dysfunction and outcome in patients with heart failure with preserved ejection fraction

**DOI:** 10.1186/1532-429X-15-S1-P268

**Published:** 2013-01-30

**Authors:** Gültekin Karakus, Beatrice A Marzluf, Diana Bonderman, Jamil Babayev, Caroline Tufaro, Stefan Pfaffenberger, Gerald Maurer, Julia Mascherbauer

**Affiliations:** 1 Cardiology, Medical University of Vienna, Vienna, Austria

## Background

Epicardial adipose tissue has been linked to cardiovascular metabolism and inflammation and has been shown to predict prevalence and progression of coronary artery disease.

The aim of the present study was to assess epicardial fat volume in patients with heart failure with preserved ejection fraction (HFPEF) in terms of quantification and predictive value.

## Methods

HFPEF was defined as serum NT-proBNP levels >220 pg/ml, E/e by echocardiography ≥8, signs or symptoms of heart failure and preserved left ventricular ejection fraction (EF≥50%).

58 HFPEF patients and 34 controls were prospectively evaluated. All patients underwent right heart catheterization. CMR studies included the assessment of cardiac function and dimensions by standard cine sequences. Epicardial fat volume was quantified offline, using dedicated software (cmr42®).

## Results

Epicardial fat volume ranged from 23 to 89ml (mean 49.3±16.2 ml; patients: 43.8±13.4 ml, controls: 58.6±16.6 ml; p<0.001). Epicardial fat volume was significantly correlated with E/e' (R=-0.37; p<0.001), NT-proBNP (R= -0.27;p=0.012), right ventricular size and function (R= -0.32; p=0.002 and R=0.40; p<0.001), left ventricular ejection fraction (R=0.36, p<0.001), left and right atrial size (R= -0.27, p=0.01; R= -0.34; p=0.001), mean pulmonary arterial pressure (R= -0.36, p=0.006), pulmonary capillary wedge pressure (R= -0.33; p=0.013), and pulmonary vascular resistance (R= -0.34; p=0.01). Epicardial fat volume was not correlated with gender, age, renal function, or body mass index.

All study participants were followed for 356±198 days. By Kaplan-Meier analysis, event-free survival was significantly worse in subjects with epicardial fat volume below the median of 43 ml (log rank p=0.038).

## Conclusions

Epicardial fat volume is inversely correlated with diastolic dysfunction, serum NT-proBNP, invasive measures of pulmonary hypertension, but not total body fat. Decreasing epicardial fat volume predicts adverse outcome in HFPEF patients. The mechanism causing decreasing epicardial fat volume in advanced disease remains to be determined.

## Funding

none

